# Comparison of accelerated and standard infliximab induction regimens in acute severe ulcerative colitis using propensity score analysis: a retrospective multicenter study in China

**DOI:** 10.1093/gastro/goae051

**Published:** 2024-06-07

**Authors:** Xinyu Liu, Hui Li, Feng Tian, Ying Xie, Xiaoqi Zhang, Min Zhi, Min Zhang, Xiaomei Song, Hong Guo, Xiaofei Li, Jie Liang, Jun Shen, Yue Li

**Affiliations:** Department of Gastroenterology, Peking Union Medical College Hospital, Chinese Academy of Medical Science & Peking Union Medical College, Beijing, P. R. China; Eight-Year Medical Doctor Program, Chinese Academy of Medical Science & Peking Union Medical College, Beijing, P. R. China; Department of Gastroenterology, Shengjing Hospital of China Medical University, Shenyang, Liaoning, P. R. China; Department of Gastroenterology, Shengjing Hospital of China Medical University, Shenyang, Liaoning, P. R. China; Department of Gastroenterology, Nanjing Drum Tower Hospital, School of Medicine, Nanjing University, Nanjing, Jiangsu, P. R. China; Department of Gastroenterology, Nanjing Drum Tower Hospital, School of Medicine, Nanjing University, Nanjing, Jiangsu, P. R. China; Department of Gastroenterology, The Sixth Affiliated Hospital, Sun Yat-sen University, Guangzhou, Guangdong, P. R. China; Department of Gastroenterology, The Sixth Affiliated Hospital, Sun Yat-sen University, Guangzhou, Guangdong, P. R. China; Department of Gastroenterology, Chongqing General Hospital, Chongqing, P. R. China; Department of Gastroenterology, Chongqing General Hospital, Chongqing, P. R. China; Department of Gastroenterology, Xijing Hospital of Digestive Diseases & State Key Laboratory of Cancer Biology, Fourth Military Medical University, Xi’an, Shaanxi, P. R. China; Department of Gastroenterology, Xijing Hospital of Digestive Diseases & State Key Laboratory of Cancer Biology, Fourth Military Medical University, Xi’an, Shaanxi, P. R. China; Department of Gastroenterology, Renji Hospital, School of Medicine, Shanghai Jiao Tong University, Shanghai, P. R. China; Department of Gastroenterology, Peking Union Medical College Hospital, Chinese Academy of Medical Science & Peking Union Medical College, Beijing, P. R. China

**Keywords:** acute severe ulcerative colitis, infliximab, accelerated induction, propensity score analysis

## Abstract

**Background:**

The optimal regimen of infliximab salvage in acute severe ulcerative colitis (ASUC) patients remains controversial. This study aimed to compare accelerated and standard infliximab induction in Chinese ASUC patients, and to explore risk factors and concrete accelerated regimens for them.

**Methods:**

Data were retrospectively collected from steroid-refractory ASUC patients receiving infliximab as rescue therapy at seven tertiary centers across China. Outcomes including colectomy and clinical remission (Mayo score ≤ 2 and every subscore ≤ 1 at Day 14) rates were compared between patients receiving accelerated and standard infliximab induction using propensity score adjustment for potential confounders. The dose–response relationship was explored by plotting restricted cubic splines. Logistic regression and Cox proportional hazards regression analyses were performed to determine risk factors for adverse outcomes. A systematic review and meta-analysis was also performed.

**Results:**

A total of 76 patients were analysed: 29 received standard and 47 received accelerated induction. The accelerated group had a higher 90-day colectomy rate (17.8% vs 0%, *P *=* *0.019) and lower clinical remission rate (27.7% vs 65.5%, *P *=* *0.001). After adjusting for propensity score and institution, there was no significant difference in colectomy or clinical remission rates (both *P *>* *0.05). Dose–effect curves showed decreased colectomy hazard with higher cumulative infliximab dosage within 5 days, with no improvement observed for increasing cumulative infliximab dosage within 28 days. Multivariate logistic regression analyses revealed C-reactive protein of >10 mg/L at infliximab initiation (odds ratio = 5.00, 95% confidence interval: 1.27–24.34) as an independent risk factor for no clinical remission. Meta-analysis also revealed no significant difference in colectomy rates at 3 months (*P *=* *0.54).

**Conclusions:**

After adjusting for confounders, there were no significant differences in colectomy or clinical remission rates between accelerated and standard infliximab induction among ASUC patients. Early administration of an intensified dosage within 5 days may be beneficial. Elevated C-reactive protein at infliximab initiation indicated need for intensive treatment.

## Introduction

Acute severe ulcerative colitis (ASUC) is a medically urgent condition of ulcerative colitis (UC) that can lead to a colectomy rate of up to 25% [1, 2] and be life-threatening. It is defined by the modified Truelove and Witts’ criteria: frequency of bloody stools (≥6 per day) and at least one sign of systemic toxicity including pulse rate of >90 b.p.m., temperature of >37.8 °C, hemoglobin of <105 g/L, and/or erythrocyte sedimentation rate (ESR) of >30 mm/h [3]. Approximately 15%–20% of UC patients will experience at least one acute severe flare during their lifetime [4]. Intravenous (IV) corticosteroids are the first-line treatment for ASUC, although 30%–40% of ASUC patients are steroid-refractory and require rescue therapy with infliximab (IFX) or cyclosporine [5]. The optimal IFX induction regimen for ASUC remains controversial. The standard IFX induction regimen of 5 mg/kg at Weeks 0, 2, and 6 was established for moderate-to-severe UC through clinical trials [6]. However, studies show enhanced IFX clearance in ASUC patients [7, 8], indicating that the standard regimen may be inadequately dosed for them. Thus, an accelerated IFX induction, defined as an intensified dose and/or shortened interval, has been proposed for ASUC in the guidelines by the British Society of Gastroenterology [9].

Although accelerated IFX induction is widely used in clinical practice, evidence supporting its use is limited. A completed Australian randomized trial (NCT02770040) has not yet been published. Existing observational studies have reached conflicting conclusions regarding the efficacy of accelerated induction [10–13]. This is likely due to small sample sizes, as well as heterogeneity in the study design and definition of ASUC. Some confounders such as disease severity also had an impact, as accelerated induction is often reserved for patients with more severe disease who often tend to have poor outcomes. Most previous studies have focused on colectomy rates as the primary outcome. However, data on the efficacy of IFX as a rescue therapy, such as clinical remission rates, are also needed, similar to clinical trials of biological agents in moderate-to-severe UC [6]. Additionally, to our knowledge, no studies have reported data on accelerated IFX induction specifically in Asian patients with ASUC.

The aims of this study were to compare short-term clinical remission and colectomy rates between standard and accelerated IFX induction regimens in patients with ASUC, using propensity score methodology to adjust for biases. We also sought to identify risk factors associated with no response to IFX treatment and explore concrete accelerated regimens, in order to help guide optimal IFX dosing strategies for ASUC.

## Patients and methods

### Study population

This multicenter retrospective cohort study enrolled consecutive patients with ASUC who met modified Truelove and Witts’ criteria [3] at seven hospitals in China [Peking Union Medical College Hospital (Beijing), Shengjing Hospital of China Medical University (Liaoning), Nanjing Drum Tower Hospital (Jiangsu), the Sixth Affiliated Hospital of Sun Yat-sen University (Guangdong), Chongqing General Hospital (Chongqing), Xijing Hospital of Digestive Diseases (Shaanxi), and Renji Hospital (Shanghai)] between December 2012 and October 2022. Eligible patients had non-response (defined as not reaching clinical response) after ≥3 days of intravenous (IV) corticosteroids treatment, and then initiated IFX rescue therapy for at least two doses. Patients were excluded if diagnosed with Crohn’s colitis, inflammatory bowel disease (IBD) unclassified, infectious colitis, concomitant immune checkpoint inhibitor-related colitis, or other non-UC colitis. Additional exclusion criteria were as follows: use of cyclosporin A or tacrolimus as rescue therapy for the current acute severe flare, admission for elective surgery, or prior IBD-related bowel resection.

Patients receiving IFX 5 mg/kg at Day 0 and again on or after Day 14 for the second dose were defined as the standard IFX induction group. There were discrepancies for the definition of accelerated induction among previous studies [10, 12, 14–17]. Generally, any regimens with higher dosage or earlier schedules than standard induction could be considered as accelerated induction. We incorporated the consideration of disease assessment within 14 days in clinical practice and defined the accelerated IFX induction group as receiving two doses of IFX on or before Day 13 and/or receiving intensified doses of ≥7.5 mg/kg.

This study was approved by the Ethical Committee of Peking Union Medical College Hospital (No. K2728).

### Data collection and processing

Patient demographics, UC history, co-morbidities, infection status, and clinical, endoscopic, and laboratory assessments throughout the acute flare, rescue therapy details, and outcomes after IFX induction were extracted through medical record review. Mayo subscores and Ulcerative Colitis Endoscopic Index of Severity (UCEIS) scores were assessed by experienced physicians. Laboratory parameters including hemoglobin, platelet count, serum albumin, C-reactive protein (CRP), and ESR were recorded at four different time points, if available: (i) start of IV steroids; (ii) first IFX infusion; (iii) first assessment after the first IFX infusion (mainly at Days 3–5), and (iv) Day 14.

Missing laboratory data were imputed by multiple imputation using a general linear model with predictive mean matching. Details regarding the multiple imputation methodology are provided in Supplementary Figure 1.

### Outcomes

The primary outcome was the 30-day colectomy rate following the first IFX infusion. Secondary outcomes included the 90-day colectomy rate, colectomy-free survival, Day 14 clinical response rate (defined as a decrease from baseline in the total Mayo score of ≥3 and ≥30%, with an accompanying decrease in the subscore for rectal bleeding of ≥1 or an absolute subscore for rectal bleeding of 0 or 1 [6]), Day 14 clinical remission rate (defined as a total Mayo score of ≤2, with each individual subscore of ≤1 [6]), length of stay, rates of severe complications (include massive gastro-intestinal bleeding, toxic megacolon, septic shock, and gastro-intestinal perforation), IFX-related adverse events [18], and IBD-related mortality.

### Systematic review and meta-analysis

We performed a systematic search in PubMed, MEDLINE, EMBASE, Cochrane Library, and Scopus databases to retrieve published articles or abstracts studying accelerated IFX on ASUC patients. The search was conducted from time of databases construction until 21 January 2024 with the following queries: (“severe*”[Title/Abstract] OR “hospital*”[Title/Abstract]) AND “colitis”[Title/Abstract] AND (“infliximab”[Title/Abstract] OR “Remicade”[Title/Abstract]) AND (“accelerat*”[All Fields] OR “intens*”[All Fields]). Manual reviews of the Annual Meeting of the European Crohn’s and Colitis Organization from 2020 to 2023 were also screened. Two study investigators independently reviewed the studies and confirmed eligibility for inclusion in the meta-analysis according to the following criteria: (i) observational or interventional study; (ii) study on patients with steroid-refractory ASUC and initiating IFX as salvage therapy; (iii) include both accelerated and standard groups, while the accelerated induction was defined in the same way as this study; and (iv) provide outcomes to calculate odds ratio (OR) and 95% confidence interval (CI) for 3-month colectomy. The exclusion criteria included as follows: (i) history of other salvage therapy in this ASUC attack; and (b) included patients with other colitis.

Q test and *I*^2^ were used to assess the heterogeneity of the included studies. A random-effect model was adopted regardless of heterogeneity to get to the conservative conclusion. Publication bias was assessed by using a funnel plot and Peters test.

### Statistical analysis

Continuous variables were expressed as means ± standard deviation (SD) or medians [interquartile range (IQR)], as appropriate. Categorical variables are expressed as numbers and percentages or frequencies. Demographic and clinical characteristics as well as outcomes were compared by using a *t*-test, Wilcoxon rank-sum test, Pearson’s chi-squared test, or the Fisher’s exact test between two groups as appropriate.

Based on clinical experience, normal values of laboratory testing, or cut-off value by receiver-operating characteristic curve (accelerated induction, clinical remission at Day 14 and colectomy at Day 90 as dependent variables, respectively) (Supplementary Figures 2–4), we transferred some continuous variables into categorical variables. Propensity score analysis was performed to predict the likelihood of receiving accelerated induction by multivariate logistic analysis (accelerated induction as the dependent variable). Outcome comparisons were adjusted for propensity score and institution (multivariate logistic or Cox regression analyses using propensity score and institution as covariates). Subgroup analyses were carried out by gender, age, body mass index (BMI), Mayo clinic subscore and UCEIS at IV steroids, days of IV steroids before IFX, and time of admission. *P*-value for interaction was calculated based on a likelihood ratio test. Sensitivity analysis was conducted by removing data from each institution. Dose–response relationships of cumulative IFX dosage with the colectomy hazard were explored by plotting restricted cubic splines [19].

Univariate and multivariate logistic regression analyses were performed to identify predictors of no clinical remission at Day 14. The multivariate model included variables with *P *<* *0.05 on univariate analysis. Cox regression analysis was performed to identify colectomy risk factors.

Two-sided *P *<* *0.05 was considered statistically significant. All statistical analyses were performed using R version 4.3.0 and IBM SPSS Statistics version 24 (IBM Corp., Armonk, NY, USA).

## Results

### Study population

**Figure 1.**
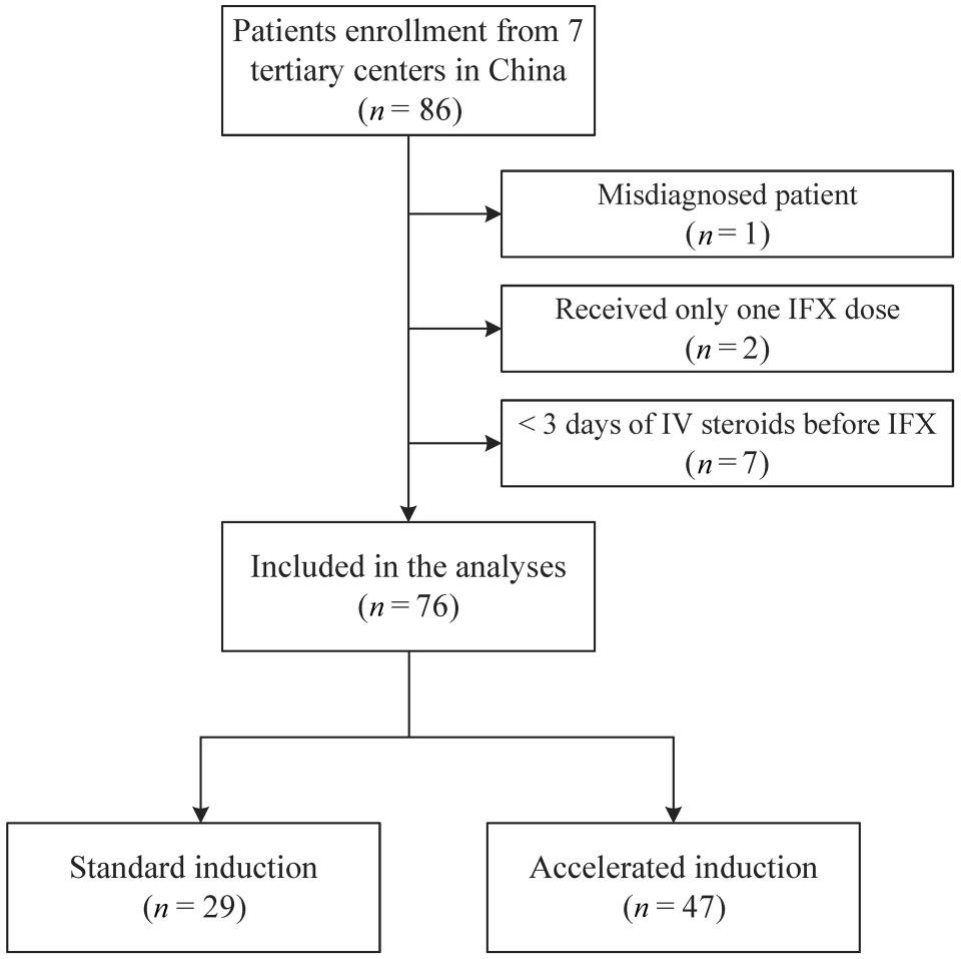
Flow diagram of patient enrollment and grouping. IFX = infliximab, IV = intravenous.

A flow chart of the study is shown in Figure 1. A total of 86 patients from the seven institutions met the inclusion criteria. After excluding 1 misdiagnosed patient, 2 who received only one IFX dose, and 7 with <3 days of IV steroids before IFX, 76 patients were finally included for analysis. Twenty-nine patients received the standard IFX induction regimen with a median time of 14 days (IQR 14–15 days) between the first and second doses. Forty-seven patients received an accelerated IFX induction regimen; 44 received early dosing (median 7 days between doses, IQR 6.5–10 days), while 3 patients received an increased dose (one 7.5 mg/kg, two 10 mg/kg). Four patients (three in the accelerated group and one in the standard group) did not finish the three doses of IFX induction: one patient underwent early colectomy, one patient was transferred to vedolizumab, one patient due to the impact of COVID-19 pandemic, and one patient due to an unknown reason (non-medical related). In the rest of the patients, the accelerated group (median 31.5 days; IQR 15.75–39.5 days) also received an earlier third dose than the standard group (median 43 days; IQR 42–44 days) (*P *<* *0.001). Seven institutions exhibited different therapeutic inclinations for IFX induction regimens (Supplementary Table 1).

### Baseline characteristics

Baseline characteristics are shown in Table 1. In general, disease severity was more severe in patients receiving accelerated induction, with higher Mayo clinical subscores (median: 9 vs 8, *P *=* *0.022) and UCEIS (median: 7 vs 6, *P *=* *0.011) at the start of IV steroids compared with patients receiving standard induction. Laboratory testing results at three different time points during induction are shown in Table 2. CRP was also higher at first IFX infusion (median: 25.90 vs 12.20 mg/L, *P *=* *0.030) and at first assessment between doses (median: 15.79 vs 4.35 mg/L, *P *=* *0.007) in the accelerated group. Other laboratory parameters including hemoglobin, platelet count, and albumin showed no difference between groups.

**Table 1.** Demographics and characteristics of study population

Variable	Standard infliximab induction (*n *=* *29)	Accelerated infliximab induction (*n *=* *47)	*P*-value
Female, *n* (%)	7 (24.1)	25 (53.2)	0.013
Age, years, mean ± SD	43.0 ± 14.9	40.0 ± 15.6	0.408
Body mass index, kg/m^2^, mean ± SD	20.4 ± 3.1	20.4 ± 3.4	0.971
Smoking status, *n* (%)			0.014
Never	18 (62.1)	41 (87.2)	
Former smoker	5 (17.2)	1 (2.1)	
Current smoker	6 (20.7)	5 (10.6)	
UC duration, years, median (IQR)	2.1 (0.7–7.2)	1.6 (0.6–3.1)	0.229
UC extent^a^, *n* (%)			0.643
Left-sided colitis (E2)	1 (3.4)	4 (8.7)	
Extensive colitis (E3)	28 (96.6)	42 (91.3)	
Co-morbidities, *n* (%)			
Autoimmune diseases	4 (13.8)	5 (10.6)	0.725
Thromboembolism	1 (3.4)	2 (4.3)	>0.999
HBV infection	1 (3.4)	2 (4.3)	>0.999
Chronic diseases	1 (3.4)	2 (4.3)	>0.999
Prior medications, *n* (%)			
5-ASAs	25 (86.2)	41 (87.2)	>0.999
Immunosuppressants	3 (10.3)	0 (0.0)	0.052
Biologics/small-molecule agents	4 (13.8)	4 (8.5)	0.472
Prior steroids, *n* (%)			0.660
No	20 (69.0)	28 (59.6)	
Yes, 1 time	5 (17.2)	17 (36.2)	
Yes, ≥2 times	4 (13.8)	2 (4.3)	
Previous IV steroids within 3 months, *n* (%)	2 (6.9)	4 (8.5)	>0.999
Mayo clinic subscore at IV steroids, *n* (%)			0.022
5	2 (6.9)	0 (0.0)	
6	0 (0.0)	2 (4.3)	
7	8 (27.6)	5 (10.6)	
8	10 (34.5)	14 (29.8)	
9	9 (31.0)	26 (55.3)	
Mayo endoscopic subscore at IV steroids^b^, *n* (%)			>0.999
2	1 (3.7)	1 (2.1)	
3	26 (96.3)	46 (97.9)	
UCEIS, median (IQR)	6 (5.5–7)	7 (6–8)	0.011
Days of IV steroids before IFX, median (IQR)	6 (4–8)	5 (4–7)	0.462
Concomitant infection before IFX induction, *n* (%)			
*Clostridium difficile*	1 (3.4)	8 (17.0)	0.141
Cytomegalovirus	0 (0.0)	2 (4.3)	0.522
Concomitant medications for infections, *n* (%)			
Broad-spectrum antibiotic	24 (82.8)	38 (80.9)	0.835
Vancomycin	1 (3.4)	17 (36.2)	0.001
Antiviral drugs	4 (13.8)	20 (42.6)	0.009

a One patient in the accelerated group received only sigmoidoscopy (no previous colonoscopy results) so the UC extent failed to be assessed.

b One patient in the standard group had suspected toxic megacolon and did not undergo colonoscopy this time.

5-ASAs = 5-aminosalicylic acid, BMI = body mass index, IFX = infliximab, IQR = interquartile range, IV = intravenous, SD = standard deviation, UC = ulcerative colitis, UCEIS = Ulcerative Colitis Endoscopic Index of Severity.

**Table 2.** Laboratory testing at IV steroids, at first IFX dose, and at first assessment between first and second IFX doses.

Variable	Standard infliximab induction (*n *=* *29)	Accelerated infliximab induction (*n *=* *47)	*P*-value
Laboratory testing at IV steroids			
Hemoglobin, g/L, mean ± SD	103 ± 27	96 ± 24	0.219
Platelet count, ×10^9^/L, mean ± SD	366 ± 140	356 ± 139	0.772
Albumin, g/L, mean ± SD	30.5 ± 4.7	31.2 ± 5.6	0.583
ESR, mm/h, median (IQR)	41 (30–74)	43.5 (29–58.5)	0.659
CRP, mg/L, median (IQR)	54.20 (35.66–97.32)	54.50 (24.00–110.22)	0.751
Laboratory testing at first IFX dose			
Hemoglobin, g/L, mean ± SD	92 ± 21	91 ± 22	0.856
Platelet count, ×10^9^/L, mean ± SD	347 ± 109	361 ± 125	0.637
Albumin, g/L, mean ± SD	30.2 ± 3.1	30.4 ± 4.7	0.808
CRP, mg/L, median (IQR)	12.20 (6.35–30.82)	25.90 (10.67–61.50)	0.030
Laboratory testing at first assessment between first and second IFX doses			
Hemoglobin, g/L, mean ± SD	99 ± 23	91 ± 19	0.126
Platelet count, ×10^9^/L, mean ± SD	330 ± 110	344 ± 156	0.695
Albumin, g/L, mean ± SD	31.4 ± 2.8	32.3 ± 4.0	0.311
CRP, mg/L, median (IQR)	4.35 (3.27–8.85)	15.79 (4.31–27.69)	0.007

CRP = C-reactive protein, ESR = erythrocyte sedimentation rate, IFX = infliximab, IQR = interquartile range, IV = intravenous, SD = standard deviation.

### Clinical outcomes of patients receiving accelerated/standard IFX induction

**Figure 2.**
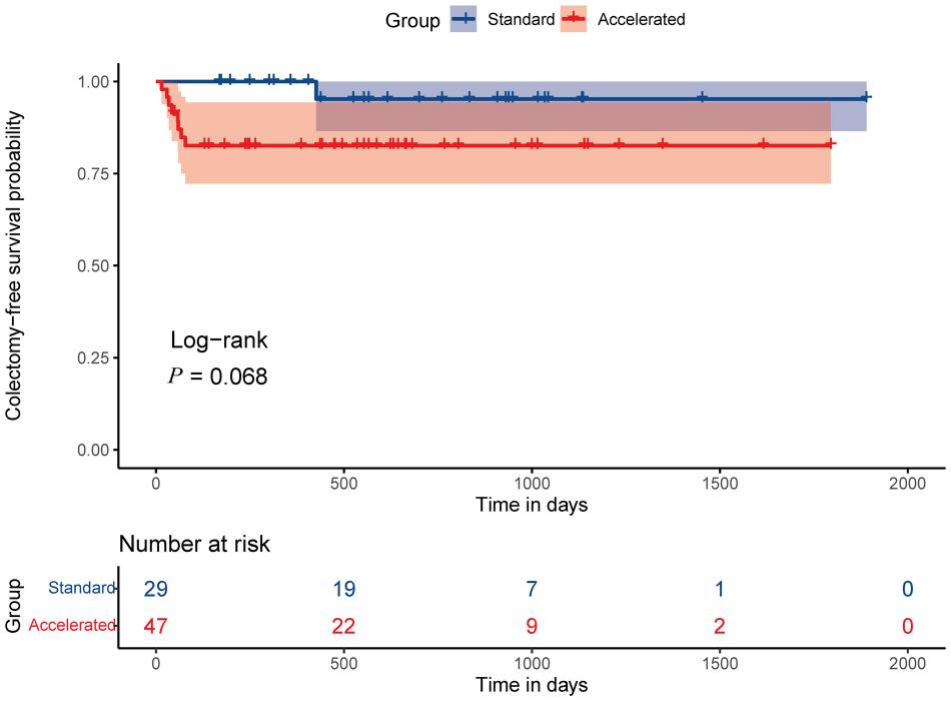
The Kaplan–Meier estimates of colectomy-free survival in acute severe ulcerative colitis patients receiving accelerated vs standard infliximab induction.

The median follow-up time was 431 days (IQR 145.5–739.5 days). Two patients in the accelerated group were lost to follow-up within 3 months due to COVID-19. In total, nine patients (11.84%) underwent colectomy at a median of 58 days (IQR 34–67 days). The 30-day colectomy rates showed no significant difference between groups (*P *=* *0.522). However, the 90-day colectomy rate was higher with accelerated dosing (17.8% vs 0%, *P *=* *0.019) (Table 3). The colectomy-free survival did not differ significantly between groups (log-rank *P *=* *0.068) (Figure 2) but, when applying more weight on the short follow-up by Gehan–Breslow–Wilcoxon test, the colectomy probability was higher in the accelerated IFX induction group (*P *=* *0.045). Clinical remission at Day 14 was significantly lower with accelerated dosing (27.7% vs 65.5%, *P *=* *0.001), while the length of hospital stay was significantly longer in the accelerated group (24 vs 15 days, *P *=* *0.001). Clinical response at Day 14 (83.0% vs 82.8%, *P *>* *0.999), severe complications (17.0% vs 6.9%, *P *=* *0.301), and IFX-related adverse events (6.4% vs 0%, *P *=* *0.283) were similar between groups (Table 3). Three patients in the accelerated group experienced IFX-related pneumonia, abnormal liver function, and infusion reaction, respectively. No IBD-related death occurred during follow-up.

**Table 3.** Clinical outcomes of standard vs accelerated infliximab induction patients

Variable	Standard infliximab induction (*n *=* *29)	Accelerated infliximab induction (*n *=* *47)	*P*-value
Colectomy at			
30 days, *n* (%)	0 (0.0)	2 (4.3)	0.522
90 days^a^, *n* (%)	0 (0.0)	8 (17.8)	0.019
Clinical response at Day 14, *n* (%)	24 (82.8)	39 (83.0)	>0.999
Clinical remission at Day 14, *n* (%)	19 (65.5)	13 (27.7)	0.001
Inpatient days, median (IQR)	15 (11.75–22.5)	24 (17–31)	0.001
Severe complications, *n* (%)	2 (6.9)	8 (17.0)	0.301
Adverse events of IFX, *n* (%)	0 (0.0)	3 (6.4)	0.283

IFX = infliximab, IQR = interquartile range.

a Two patients in the accelerated group were lost to follow-up within 3 months due to COVID-19, so the denominator was 45.

### Propensity score analysis

Univariate and multivariate logistic regression analyses were performed to explore predictors of receiving accelerated IFX induction. After removing some collinear variables, those with statistical significance were included in the multivariate logistic regression model incorporating gender, Mayo clinical subscore, UCEIS and hemoglobin at IV steroids, Mayo clinical subscore and CRP at first IFX dose, and concomitant use of vancomycin and antiviral drugs (Supplementary Table 2). The independent predictive factors for accelerated IFX induction included Mayo clinical subscore at first IFX dose (*P *=* *0.04), and concomitant use of vancomycin (*P *=* *0.02). After adjusting for propensity score and institution, there remained no difference between accelerated and standard induction for colectomy [hazard ratio (HR)* *=* *0.13, 95% CI: 0.01–2.91, *P *=* *0.20] and no clinical remission at Day 14 (OR = 1.73, 95% CI: 0.38–7.84, *P *=* *0.48).

### Subgroup and sensitivity analyses

**Figure 3.**
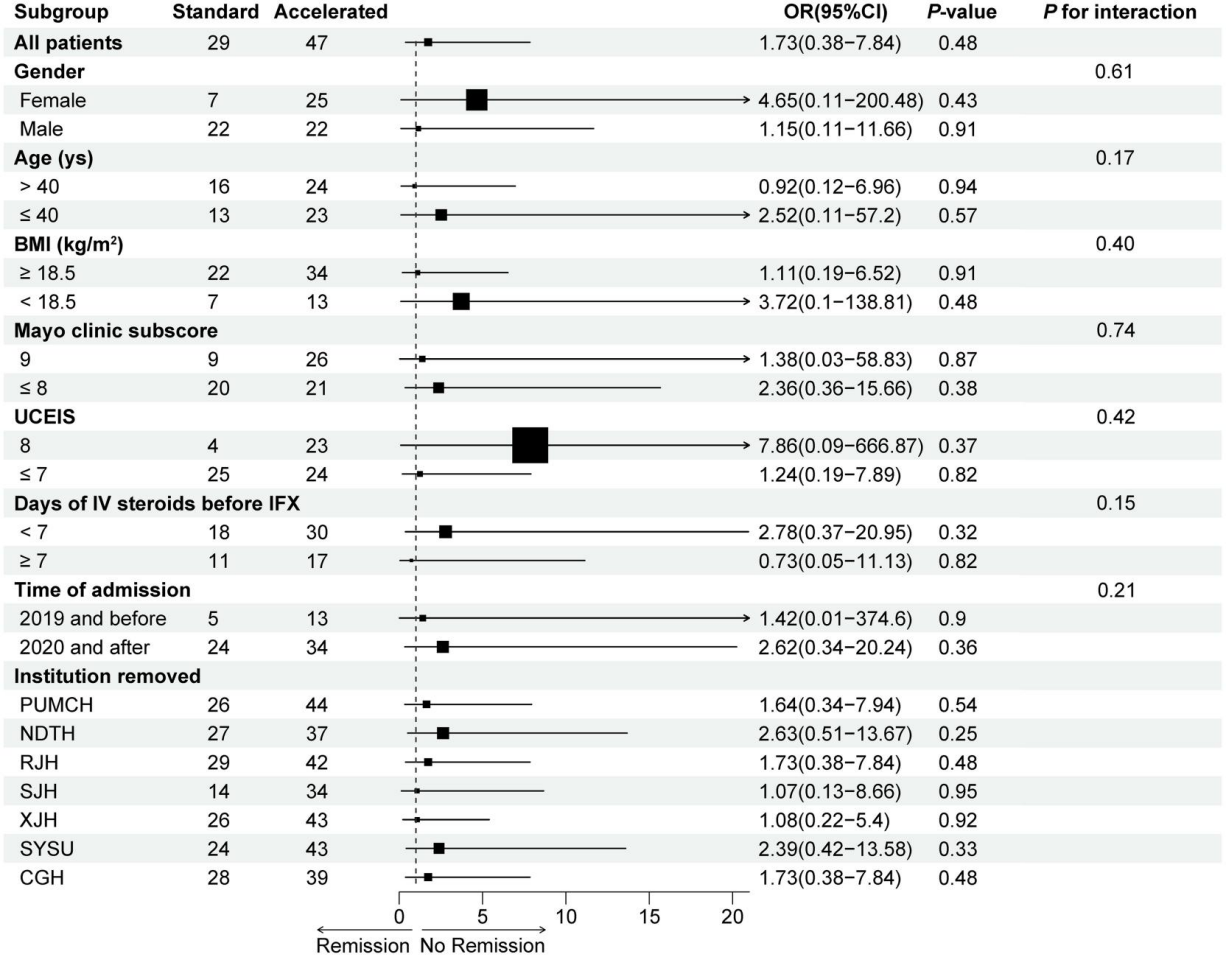
Forest plot of subgroup and sensitivity analyses for the impact of accelerated infliximab induction on no clinical remission risk at Day 14 (adjusted for propensity score and institution). BMI = Body mass index, IFX = infliximab, IV = intravenous, UCEIS = Ulcerative Colitis Endoscopic Index of Severity.

We further conducted subgroup and sensitivity analyses to investigate whether the relationship between IFX regimens and no clinical remission rate varied by different gender, age, BMI, disease severity, IV steroids time, year of admission, and institution. There was no significant interaction detected for any variable (*P *>* *0.05) and the removal of data from any institution did not change the results significantly (Figure 3).

### Dose–effect curves of cumulative IFX dosage with colectomy hazard

**Figure 4.**
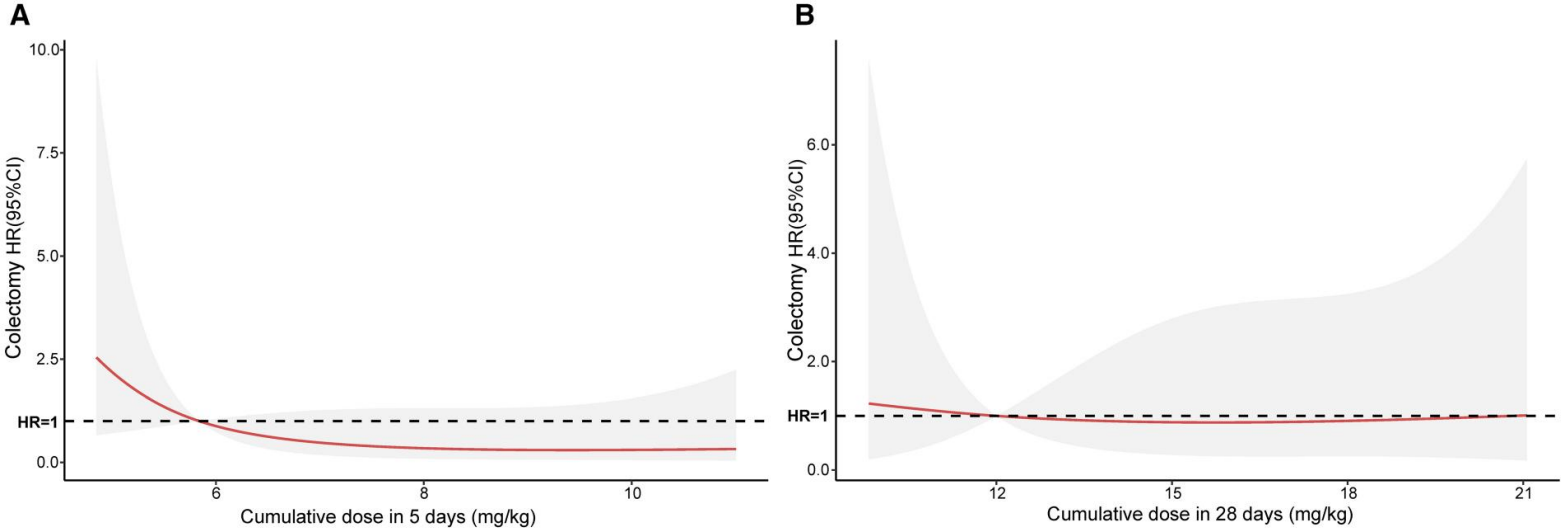
Association between cumulative infliximab dosage and colectomy hazard. (A) Dose–response curve for the association between cumulative infliximab dosage within 5 days and colectomy hazard (adjusted for propensity score and institution). (B) Dose–response curve for the association between cumulative infliximab dosage within 28 days and colectomy hazard (adjusted for propensity score and institution). HR = hazard ratio.

Given the fact that the actual doses that patients received were continuous variables in real clinical practice, we calculated the precise doses using the weight and performed dose–effect analyses to investigate the relationship of cumulative IFX dosage with colectomy hazard. Interestingly, the colectomy hazard decreased with higher cumulative IFX dosage within 5 days and remained stable at ∼8 mg/kg (Figure 4A). However, for cumulative dosage within 28 days, no improvement was observed regardless of the dosage administered (Figure 4B).

### Factors associated with no remission and colectomy

#### No clinical remission at Day 14

**Figure 5.**
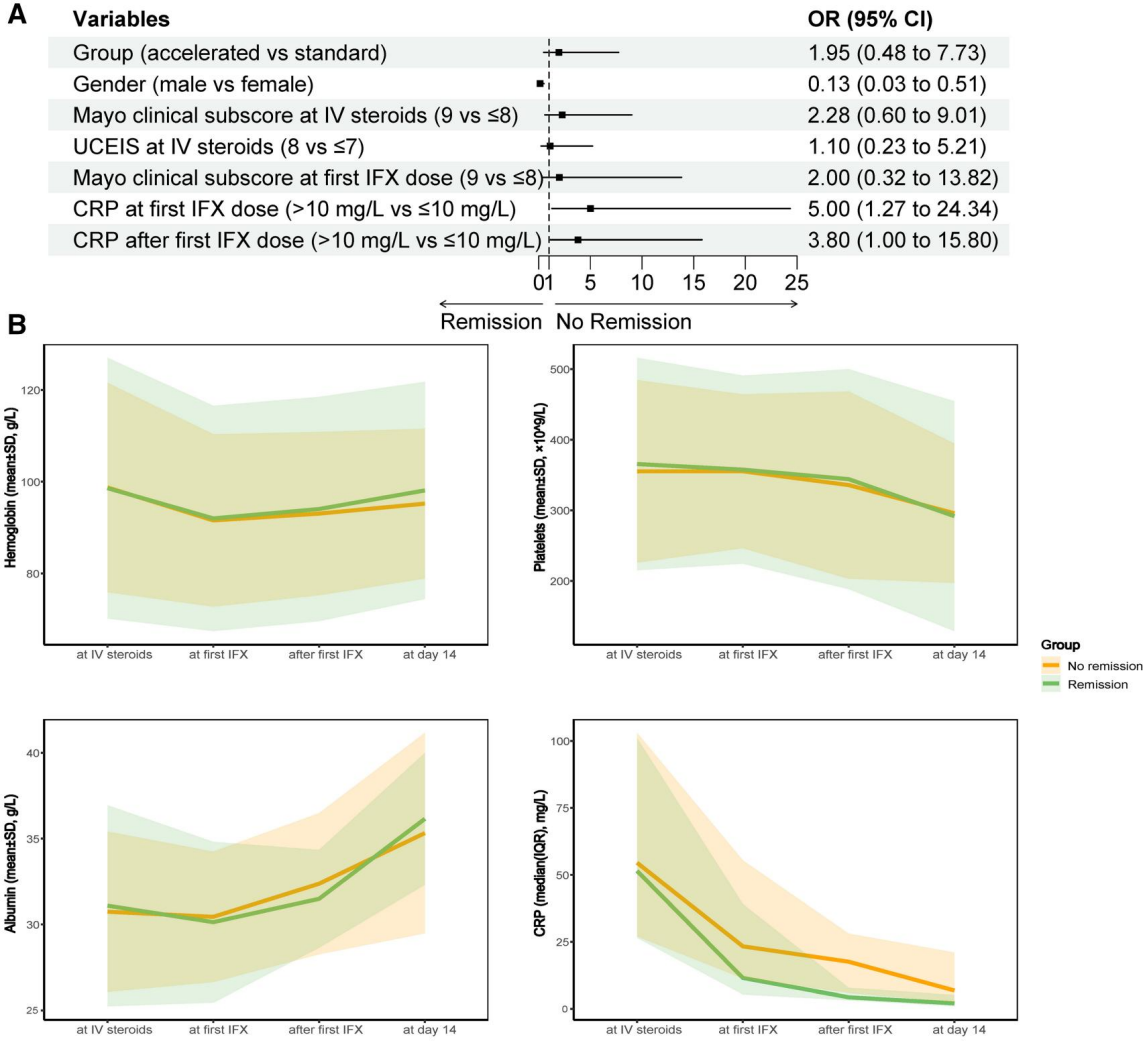
CRP might be an important indicator for predicting no clinical remission. (A) Forest plot of multivariate logistic regression model predicting no clinical remission at Day 14. (B) Line charts of laboratory test results (hemoglobin, platelet count, serum albumin, and CRP) for patients with and without clinical remission at Day 14 through the induction. CRP = C-reactive protein, IFX = infliximab, SD = standard deviation.

We performed univariate and multivariate logistic analyses to explore risk factors for no clinical remission (Supplementary Table 3). Female gender (OR = 7.69, 95% CI: 1.96–33.33, *P *=* *0.01) and CRP of >10 mg/L at first IFX dose (OR = 5.00, 95% CI: 1.27–24.34, *P *=* *0.03) were associated with a higher risk of no clinical remission at Day 14 (Figure 5A). CRP of >10 mg/L after first IFX (OR = 3.80, 95% CI: 0.996–15.80, *P *=* *0.054) was borderline significant.

#### Colectomy

On univariate Cox regression analysis, UC duration of >10 years, concomitant chronic diseases, concomitant *Clostridioides difficile* infection after IFX induction, Mayo rectal bleeding subscore at first IFX, Mayo rectal bleeding and total clinical subscore at Day 14, and concomitant use of vancomycin were risk factors for colectomy (Supplementary Table 4). However, limited colectomy cases precluded multivariate analysis.

### Dynamic change in hemoglobin, platelet count, albumin, and CRP through IFX induction

We drew line charts of laboratory test results for patients with and without clinical remission at Day 14. There was no significant difference in hemoglobin, platelet count, and albumin overall, while patients who failed to achieve clinical remission exhibited higher CRP levels since the first induction of IFX (Figure 5B).

### Systematic review and meta-analysis

**Figure 6.**
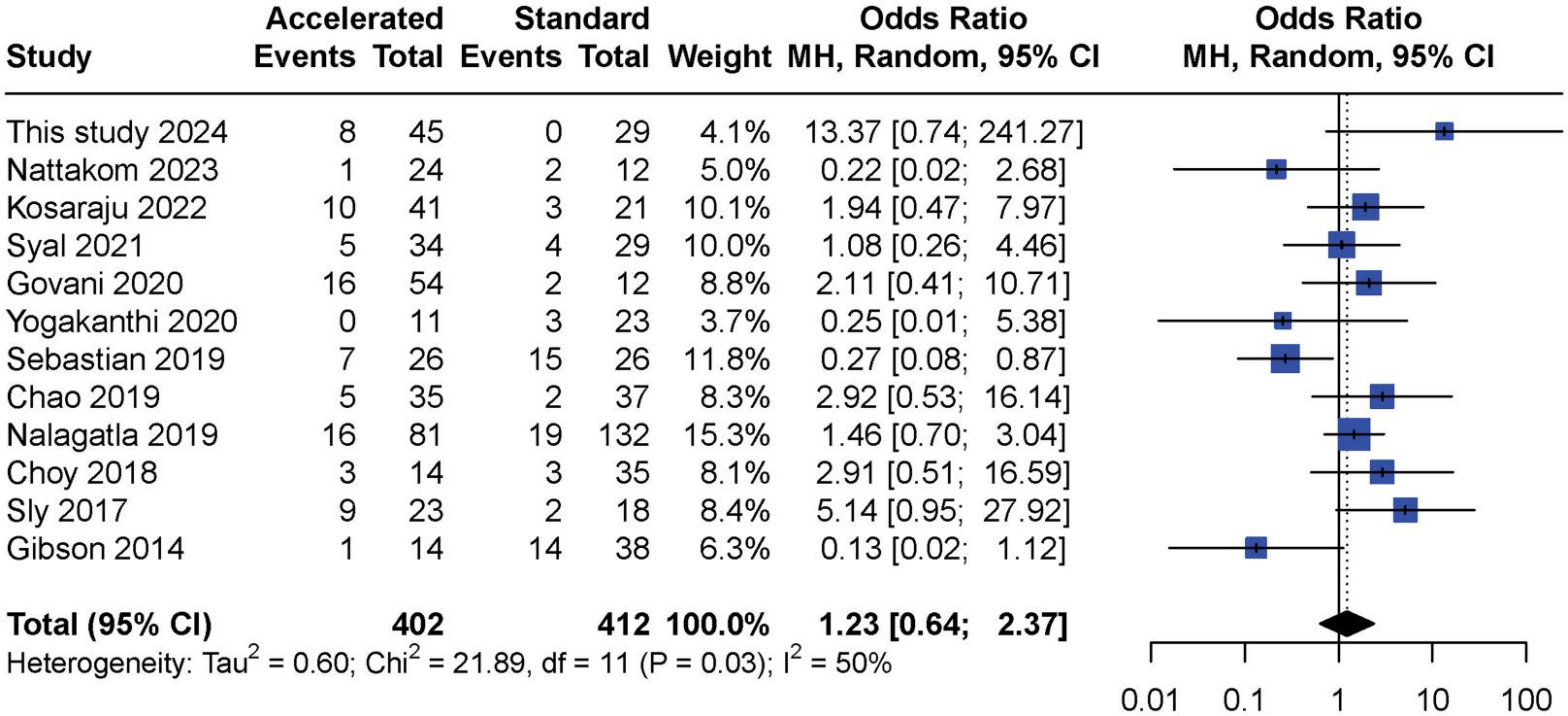
Forest plot of meta-analysis of colectomy rates at 3 months with standard and accelerated infliximab induction for acute severe ulcerative colitis.

The inclusion of literature is schemed in Supplementary Figure 5. A total of 12 eligible studies were included in the meta-analysis, with 402 patients in the accelerated group and 412 patients in the standard group (Table 4) [12, 13, 16, 17, 20–26]. There was significant heterogeneity between the studies (*P *=* *0.03, *I*^2^* *=* *50%). According to the random effects model, there was no significant difference in colectomy rates at 3 months (OR = 1.23, 95% CI: 0.64–2.37, *P *=* *0.54) (Figure 6), which showed the same conclusion with most of the individual studies except for the work by Sebastian *et al.* [17]. The funnel plot showed a symmetrical pattern in general, which indicated no significant publication bias (*P *=* *0.41) (Supplementary Figure 6).

**Table 4.** Information on literature in meta-analysis

Author	Year	Country	Full text or abstract	Number of patients in accelerated group	Number of patients in standard group
Gibson *et al*.	2014	Ireland	Abstract	14	38
Sly *et al*.	2017	USA	Abstract	23	18
Choy *et al*.	2018	Australia	Full text	14	35
Nalagatla *et al*.	2019	USA	Full text	81	132
Chao *et al*.	2019	Canada	Full text	35	37
Sebastian *et al*.	2019	UK	Full text	26	26
Yogakanthi *et al*.	2020	Australia	Abstract	11	23
Govani *et al*.	2020	USA	Full text	54	12
Syal *et al*.	2021	USA	Full text	34	29
Kosaraju *et al*.	2022	USA	Full text	41	21
Nattakom *et al*.	2023	USA	Abstract	24	12
Liu *et al*. (this study)	2024	China	Full text	45	29

## Discussion

In this multicenter retrospective study, we found no significant difference in short-term clinical remission rates and colectomy-free survival between ASUC patients who received standard vs accelerated IFX induction therapy, after controlling for confounding factors using propensity score analysis. This conclusion was further validated by using a meta-analysis. The dose–effect curves revealed that an enhanced infliximab dosage within 5 days but not 28 days might decrease colectomy risk. We also found some risk factors that can predict prognosis in ASUC patients effectively, including CRP levels at and after IFX initiation. There were also gaps between these actual risk factors and the factors that physicians considered when making treatment decisions in practice. This provides a basis for modifying future clinical decision-making.

We observed higher risks of no clinical remission at Day 14 and short-term colectomy in patients receiving accelerated IFX dosing. However, after adjusting for covariates, there was no significant difference between the two groups in either outcome. This is in accordance with the conclusions of most other studies, including two meta-analyses [12, 14]. Given the fact that some ASUC patients did have enhanced IFX clearance [7, 8], accelerated induction was supposed to help to some extent. The counterproductive results may have two underlying reasons. First, the identification of patients who have the potential to benefit from accelerated regimens has not been successful enough. Several studies have proposed that patients with more severe disease (often means more severe IFX loss) are more likely to benefit from accelerated induction therapy [17, 27]. Also, the various mechanisms of UC [28, 29] also indicate primary resistance to IFX in some patients no matter which regimens we adopted. More thorough research studying the biomarkers for predicting IFX efficacy as well as therapeutic drug monitoring are warranted. Second, the IFX accelerated induction strategy may not be enough in existing studies. We found an interesting phenomenon that intensified dosage within 5 days could reduce colectomy hazard but would not have beneficial effects when it comes to 28 days. It indicated that the early escalation of dosage might be crucial in preventing disease progression. The definition of accelerated IFX induction may require updating and reassessment in comparison with standard induction. Additionally, it is possible that certain important confounding factors may influence the dose–effect relationship. Therefore, we should approach this finding with caution, and the optimal regimen should be further explored within different subgroups.

In our study, it seemed that, compared with different IFX induction strategies, other factors may be more relevant and predictive of different prognoses. Different studies have identified risk factors for unsuccessful IFX induction. In addition to baseline Mayo endoscopic activity [16], some laboratory tests were widely reported as important indicators. Higher CRP [15], lower albumin [10, 13, 15], and lower hemoglobin [13], especially the former two around IFX initiation, were all related to higher risk of colectomy or induction failure. Some secondary indicators such as the CRP/albumin ratio may also help to identify high-risk colectomy patients [16].

In our study, we found that CRP of >10 mg/L around the first IFX dose may be an important indicator of no clinical remission with IFX induction. The CRP level is also a predictive factor for intravenous corticosteroid resistance in ASUC patients [30], which emphasizes the importance of CRP for physicians to treat ASUC. For patients with higher CRP, intensified treatment such as accelerated IFX induction should be considered. At the same time, based on the finding that accelerated induction failed to lower colectomy risk, these patients should convert to surgery quickly if needed. However, neither albumin nor hemoglobin was found to be related to the outcome in our study. We also found female gender to be an independent predictor of lower probability of clinical remission but, after reviewing the literature, we found that most studies did not report a difference by gender. Shah *et al.* [11] reported being female as a predictive factor for needing accelerated IFX dosing, while another study found that males were less likely to achieve clinical remission during tumor necrosis factor inhibitors induction for UC [31]. Therefore, we attributed this to a bias in our study population, in which females showed higher disease activity. As for risk factors for colectomy, univariate Cox regression analysis provided some clues that, in addition to the disease severity represented by the Mayo score, long UC duration (>10 years), concomitant chronic diseases, and *C. difficile* infection may also be related to an increased probability of colectomy.

Through propensity score analysis, we found some variables related to accelerated IFX induction which may reflect factors that physicians consider when making decisions on the treatment strategy. However, there seemed to be some distinction between these and the actual risk factors for poor prognosis. Physicians tended to place more weight on clinical severity. Additionally, some individual preferences mattered, given that patients receiving empirical anti-infective therapy were more likely to receive accelerated IFX induction due to the active treatment approach. This gap suggests that we should pay more attention to objective indicators such as CRP when determining induction strategies.

From another perspective, the diagnostic criteria for ASUC should be reconsidered, which would impact patient enrollment and the conclusions of similar studies. Although CRP was found to correlate with severity and prognosis in ASUC patients, the most widely used modified Truelove and Witts’ criteria, also adopted in our study, do not include CRP levels. The guideline from the British Society of Gastroenterology has proposed including CRP of >30 mg/L as a diagnostic criterion for ASUC [9]. Additionally, it is worth discussing whether endoscopic assessment should be considered for inclusion in the diagnostic criteria. Re-evaluating the ASUC criteria to potentially incorporate CRP levels and endoscopic findings could improve physicians’ awareness and allow stronger conclusions in future ASUC studies.

Our study has some limitations. First, although we enrolled patients from multiple centers across China, the sample size was still limited. The cases that strictly met the ASUC criteria accounted for only a small proportion of UC patients. Second, different physicians at different hospitals adopted accelerated induction based on their own criteria, which introduced substantial heterogeneity and subjectivity that may limit generalizability. Third, several patients were lost to follow-up in the first 3 months due to the impact of COVID-19.

## Conclusions

Patients receiving accelerated IFX induction exhibited a higher degree of disease activity. However, after adjusting for confounding factors using a propensity score method, there was no significant difference in the clinical remission and colectomy rates between patients with different induction strategies. Nevertheless, the intensified IFX dosage administered within 5 days appeared to decrease the colectomy hazard. A higher level of CRP around IFX initiation was an important indicator for the lack of clinical remission. Additionally, certain other factors, including higher Mayo score, longer duration of UC, concomitant chronic diseases, and *C. difficile* infection, may be associated with an increased risk of colectomy. These objective variables should be given more consideration by physicians when making clinical decisions.

## Supplementary Material

goae051_Supplementary_Data
