# Supplementary material for: Comparison of accelerated and standard infliximab induction regimens in acute severe ulcerative colitis using propensity score analysis: a retrospective multicenter study in China
Source: Gastroenterol Rep (Oxf). 2024 Jun 7;12:goae051. doi: 10.1093/gastro/goae051 (PMC11162152; doi:10.1093/gastro/goae051)
Supplement: goae051_Supplementary_Data [file goae051_supplementary_data.docx]

**Supplementary Table 1.** Infliximab induction regimens in different institutions.

| Hospital | Standard infliximab induction, *n* (%) | Accelerated infliximab induction, *n* (%) | Increased dose | Days between 1^st^ and 2^nd^ doses, median (IQR) |
| --- | --- | --- | --- | --- |
| Peking Union Medical College Hospital (*n* = 6) | 3 (50.0) | 3 (50.0) | None | 10.5 (7–14) |
| Nanjing Drum Tower Hospital (*n* = 12) | 2 (16.7) | 10 (83.3) | One 7.5 mg/kg | 7.5 (5.75–12.5) |
| Renji Hospital (*n* = 5) | 0 (0.0) | 5 (100.0) | One 10 mg/kg | 7 (7–7) |
| Shengjing Hospital of China Medical University (*n* = 28) | 15 (53.6) | 13 (46.4) | None | 14 (7–14) |
| Xijing Hospital of Digestive Diseases (*n* = 7) | 3 (42.9) | 4 (57.1) | None | 9 (7–16) |
| The Sixth Affiliated Hospital of Sun Yat-sen University (*n* = 9) | 5 (55.6) | 4 (44.4) | One 10 mg/kg | 14 (13–14) |
| Chongqing General Hospital (*n* = 9) | 1 (11.1) | 8 (88.9) | None | 10 (8–12) |

IQR, interquartile range.

**Supplementary Table 2.** Propensity score model by logistic regression analysis of the predictive factors for accelerated IFX induction

| Variables | Univariable analysis | | Multivariable analysis | |
| --- | --- | --- | --- | --- |
|  | OR (95%CI) | *P-*value | OR (95%CI) | *P-*value |
| Gender (male vs female) | 0.28 (0.10–0.78) | 0.01 | 0.31 (0.08–1.12) | 0.08 |
| Age (>40 years vs ≤40 years) | 0.85 (0.34–2.14) | 0.73 | - | - |
| Body mass index (≥18.5 kg/m^2^ vs <18.5 kg/m^2^) | 0.83 (0.29–2.40) | 0.74 | - | - |
| UC duration (>10 years vs ≤10 years) | 0.26 (0.06–1.13) | 0.07 | - | - |
| UC extent (extensive colitis vs left-sided colitis) | 0.38 (0.04–3.57) | 0.40 | - | - |
| Concomitant autoimmune diseases (yes vs no) | 0.74 (0.18–3.01) | 0.68 | - | - |
| Concomitant thromboembolism (yes vs no) | 1.24 (0.11–14.19) | 0.86 | - | - |
| Concomitant *hepatitis B virus* infection (yes vs no) | 1.24 (0.11–14.19) | 0.86 | - | - |
| Concomitant chronic diseases (yes vs no) | 1.24 (0.11–14.19) | 0.86 | - | - |
| Concomitant *Clostridium difficile* infection before IFX induction (yes vs no) | 5.74 (0.69–48.04) | 0.11 | - | - |
| Concomitant cytomegalovirus infection before IFX induction (yes vs no) | NA | NA | NA | NA |
| Prior 5-ASAs (yes vs no) | 1.09 (0.28–4.23) | 0.90 | - | - |
| Prior immunosuppressants (yes vs no) | NA | NA | NA | NA |
| Prior biologics/small-molecule agents (yes vs no) | 0.58 (0.13–2.51) | 0.47 | - | - |
| Prior steroids (yes vs no) | 1.00 (0.48–2.05) | 0.99 | - | - |
| Prior IV steroids within 3 months (yes vs no) | 1.26 (0.22–7.27) | 0.80 | - | - |
| Mayo subscore, stool frequency, at IV steroids (3 vs ≤2) | 2.37 (0.8–7.03) | 0.12 | - | - |
| Mayo subscore, rectal bleeding, at IV steroids (3 vs ≤2) | 1.98 (0.99–3.98) | 0.06 | - | - |
| Mayo subscore, physician’s global assessment, at IV steroids (3 vs ≤2) | 1.72 (0.40–7.43) | 0.47 | - | - |
| Mayo clinical subscore at IV steroids (9 vs ≤8) | 1.78 (1.07–2.96) | 0.03 | 1.32 (0.59–3.10) | 0.51 |
| Mayo endoscopic subscore at IV steroids (3 vs 2) | 1.64 (0.10–26.94) | 0.73 | - | - |
| UCEIS at IV steroids (8 vs ≤7) | 1.65 (1.12–2.42) | 0.01 | 1.64 (0.96–2.96) | 0.08 |
| Hemoglobin at IV steroids (>105 g/L vs ≤105 g/L) | 0.33 (0.13–0.86) | 0.02 | 0.40 (0.10–1.50) | 0.18 |
| PLT at IV steroids (>400×10^9^/L vs ≤400×10^9^/L) | 0.52 (0.20–1.36) | 0.19 | - | - |
| Albumin at IV steroids (>28 g/L vs ≤28 g/L) | 1.44 (0.55–3.81) | 0.46 | - | - |
| Erythrocyte sedimentation rate at IV steroids (>15 mm/h vs ≤15 mm/h) | 3.06 (0.68–13.8) | 0.15 | - | - |
| CRP at IV steroids (>100 mg/L vs ≤100 mg/L) | 1.98 (0.67–5.81) | 0.22 | - | - |
| Mayo subscore, stool frequency, at first IFX dose (3 vs ≤2) | 2.20 (1.09–4.44) | 0.03 | - | - |
| Mayo subscore, rectal bleeding, at first IFX dose (3 vs ≤2) | 2.18 (1.26–3.78) | 0.01 | - | - |
| Mayo subscore, physician’s global assessment, at first IFX dose (3 vs ≤2) | 1.90 (0.80–4.50) | 0.15 | - | - |
| Mayo clinical subscore at first IFX dose (9 vs ≤8) | 1.48 (1.11–1.97) | 0.01 | 1.53 (1.04–2.37) | 0.04 |
| Hemoglobin at first IFX dose (>105 g/L vs ≤105 g/L) | 0.46 (0.15–1.35) | 0.16 | - | - |
| PLT at first IFX dose (>300×10^9^/L vs ≤300×10^9^/L) | 1.37 (0.53–3.53) | 0.52 | - | - |
| Albumin at first IFX dose (>30 g/L vs ≤30 g/L) | 1.75 (0.69–4.45) | 0.24 | - | - |
| CRP at first IFX dose (>50 mg/L vs ≤50 mg/L) | 5.38 (1.43–20.24) | 0.01 | 2.01 (0.38–12.48) | 0.42 |
| Concomitant use of wide-spectrum antibiotics (yes vs no) | 0.88 (0.26–2.92) | 0.83 | - | - |
| Concomitant use of vancomycin (yes vs no) | 15.87 (2.00–125.85) | 0.01 | 3.80 (3.37–1508.86) | 0.02 |
| Concomitant use of antiviral drugs (yes vs no) | 4.63 (1.4–15.33) | 0.01 | 2.69 (0.49–17.88) | 0.27 |

5-ASAs, 5-aminosalicylic acid; CRP, C-reactive protein; IFX, infliximab; IV, intravenous; NA, not applicable; PLT, platelet; UC, ulcerative colitis; UCEIS, Ulcerative Colitis Endoscopic Index of Severity; OR, odds ratio; CI, confidence interval.

**Supplementary Table 3.** Univariable and multivariable logistic regression analysis of the predictive factors for no clinical remission at day 14

| Variables | Univariable analysis | | Multivariable analysis | |
| --- | --- | --- | --- | --- |
|  | OR (95%CI) | *P-*value | OR (95%CI) | *P-*value |
| Group (accelerated vs standard) | 4.97 (1.84–13.40) | 0.002 | 1.95 (0.48–7.73) | 0.34 |
| Gender (male vs female) | 0.16 (0.05–0.46) | <0.001 | 0.13 (0.03–0.51) | 0.01 |
| Age (>40 years vs ≤40 years) | 0.97 (0.39–2.39) | 0.94 | - | - |
| Body mass index (≥18.5 kg/m^2^ vs <18.5 kg/m^2^) | 0.89 (0.32–2.50) | 0.82 | - | - |
| UC duration (>10 years vs ≤10 years) | 0.54 (0.13–2.18) | 0.39 | - | - |
| UC extent (extensive colitis vs left-sided colitis) | 0.91 (0.14–5.74) | 0.92 | - | - |
| Concomitant autoimmune diseases (yes vs no) | 0.90 (0.22–3.62) | 0.88 | - | - |
| Concomitant thromboembolism (yes vs no) | 1.48 (0.13–16.81) | 0.75 | - | - |
| Concomitant *hepatitis B virus* infection (yes vs no) | 1.48 (0.13–16.81) | 0.75 | - | - |
| Concomitant chronic diseases (yes vs no) | 0.35 (0.03–3.97) | 0.40 | - | - |
| Concomitant *Clostridium difficile* infection before IFX induction (yes vs no) | 0.90 (0.22–3.62) | 0.88 | - | - |
| Concomitant cytomegalovirus infection before IFX induction (yes vs no) | 0.72 (0.04–11.8) | 0.82 | - | - |
| Concomitant *Clostridium difficile* infection after IFX induction (yes vs no) | 1.48 (0.13–16.81) | 0.75 | - | - |
| Concomitant cytomegalovirus infection after IFX induction (yes vs no) | NA | NA | NA | NA |
| Prior 5-ASAs (yes vs no) | 3.83 (0.91–16.05) | 0.07 | - | - |
| Prior immunosuppressants (yes vs no) | NA | NA | NA | NA |
| Prior biologics/small-molecule agents (yes vs no) | 2.37 (0.45–12.48) | 0.31 | - | - |
| Prior steroids (yes vs no) | 1.37 (0.66–2.87) | 0.40 | - | - |
| Prior IV steroids within 3 months (yes vs no) | 0.71 (0.13–3.72) | 0.68 | - | - |
| Mayo subscore, stool frequency, at IV steroids (3 vs ≤2) | 4.56 (1.11–18.7) | 0.04 | - | - |
| Mayo subscore, rectal bleeding, at IV steroids (3 vs ≤2) | 2.04 (0.81–5.13) | 0.13 | - | - |
| Mayo subscore, physician’s global assessment, at IV steroids (3 vs ≤2) | 12.04 (1.41–102.49) | 0.02 | - | - |
| Mayo clinical subscore at IV steroids (9 vs ≤8) | 3.69 (1.4–9.76) | 0.01 | 2.28 (0.60–9.01) | 0.22 |
| Mayo endoscopic subscore at IV steroids (3 vs 2) | NA | NA | NA | NA |
| UCEIS at IV steroids (8 vs ≤7) | 3.96 (1.37–11.44) | 0.01 | 1.10 (0.23–5.21) | 0.90 |
| Hemoglobin at IV steroids (>105 g/L vs ≤105 g/L) | 0.71 (0.29–1.79) | 0.47 | - | - |
| PLT at IV steroids (>350×10^9^/L vs ≤350×10^9^/L) | 0.62 (0.25–1.56) | 0.32 | - | - |
| Albumin at IV steroids (>30 g/L vs ≤30 g/L) | 0.78 (0.31–1.93) | 0.59 | - | - |
| Erythrocyte sedimentation rate at IV steroids (>35 mm/h vs ≤35 mm/h) | 0.57 (0.23–1.46) | 0.25 | - | - |
| CRP at IV steroids (>80 mg/L vs ≤80 mg/L) | 1.39 (0.55–3.50) | 0.49 | - | - |
| Mayo subscore, stool frequency, at first IFX dose (3 vs ≤2) | 7.42 (2.65–20.77) | <0.001 | - | - |
| Mayo subscore, rectal bleeding, at first IFX dose (3 vs ≤2) | 2.64 (1.02–6.82) | 0.05 | - | - |
| Mayo subscore, physician’s global assessment, at first IFX dose (3 vs ≤2) | 5.79 (2.06–16.23) | <0.001 | - | - |
| Mayo clinical subscore at first IFX dose (9 vs ≤8) | 7.00 (2.12–23.16) | <0.001 | 2.00 (0.32–13.82) | 0.46 |
| Hemoglobin at first IFX dose (>105 g/L vs ≤105 g/L) | 0.57 (0.19–1.67) | 0.31 | - | - |
| PLT at first IFX dose (>300×10^9^/L vs ≤300×10^9^/L) | 1.32 (0.52–3.37) | 0.56 | - | - |
| Albumin at first IFX dose (>30 g/L vs ≤30 g/L) | 1.24 (0.50–3.08) | 0.64 | - | - |
| CRP at first IFX dose (>10 mg/L vs ≤10 mg/L) | 4.66 (1.62–13.46) | 0.005 | 5.00 (1.27–24.34) | 0.03 |
| Hemoglobin after first IFX dose (>75 g/L vs ≤75 g/L) | 2.36 (0.82–6.75) | 0.11 | - | - |
| PLT after first IFX dose (>400×10^9^/L vs ≤400×10^9^/L) | 0.83 (0.31–2.23) | 0.71 | - | - |
| Albumin after first IFX dose (>30 g/L vs ≤30 g/L) | 1.12 (0.43–2.94) | 0.81 | - | - |
| CRP after first IFX dose (>10 mg/L vs ≤10 mg/L) | 6.25 (2.22–17.57) | <0.001 | 3.80 (0.996–15.80) | 0.054 |
| Concomitant use of wide-spectrum antibiotics (yes vs no) | 2.11 (0.66–6.80) | 0.21 | - | - |
| Concomitant use of vancomycin (yes vs no) | 1.19 (0.41–3.49) | 0.75 | - | - |
| Concomitant use of antiviral drugs (yes vs no) | 0.80 (0.30–2.11) | 0.65 | - | - |

5-ASAs, 5-aminosalicylic acid; CRP, C-reactive protein; IFX, infliximab; IV, intravenous; NA, not applicable; PLT, platelet; UC, ulcerative colitis; UCEIS, Ulcerative Colitis Endoscopic Index of Severity; OR, odds ratio; CI, confidence interval.

**Supplementary Table 4.** Univariate cox regression analysis of the predictive factors for colectomy

| Variables | Univariable analysis | |
| --- | --- | --- |
|  | HR (95%CI) | *P-*value |
| Group (accelerated vs standard) | 5.57 (0.70–44.50) | 0.11 |
| Gender (male vs female) | 0.94 (0.25–3.49) | 0.92 |
| Age (>40 years vs ≤40 years) | 1.86 (0.46–7.42) | 0.38 |
| Body mass index (≥18.5 kg/m^2^ vs <18.5 kg/m^2^) | 3.14 (0.39–25.10) | 0.28 |
| UC duration (>10 years vs ≤10 years) | 4.08 (1.02–16.3) | 0.047 |
| UC extent (extensive colitis vs left-sided colitis) | NA | NA |
| Concomitant autoimmune diseases (yes vs no) | NA | NA |
| Concomitant thromboembolism (yes vs no) | 4.68 (0.58–38.10) | 0.15 |
| Concomitant *hepatitis B virus* infection (yes vs no) | NA | NA |
| Concomitant chronic diseases (yes vs no) | 15.10 (2.98–76.50) | 0.001 |
| Concomitant *Clostridium difficile* infection before IFX induction (yes vs no) | 2.06 (0.43–9.91) | 0.37 |
| Concomitant cytomegalovirus infection before IFX induction (yes vs no) | NA | NA |
| Concomitant *Clostridium difficile* infection after IFX induction (yes vs no) | 8.08 (1.66–39.40) | 0.01 |
| Concomitant cytomegalovirus infection after IFX induction (yes vs no) | NA | NA |
| Prior 5-ASAs (yes vs no) | 1.23 (0.15–9.81) | 0.85 |
| Prior immunosuppressants (yes vs no) | 2.85 (0.36–22.80) | 0.32 |
| Prior biologics/small-molecule agents (yes vs no) | 1.08 (0.14–8.62) | 0.95 |
| Prior steroids (yes vs no) | 1.26 (0.49–3.23) | 0.63 |
| Prior IV steroids within 3 months (yes vs no) | 1.54 (0.19–12.30) | 0.69 |
| Mayo subscore, stool frequency, at IV steroids (3 vs ≤2) | 0.62 (0.13–2.99) | 0.55 |
| Mayo subscore, rectal bleeding, at IV steroids (3 vs ≤2) | 7.46 (0.93–59.70) | 0.06 |
| Mayo subscore, physician’s global assessment, at IV steroids (3 vs ≤2) | 0.42 (0.09–2.02) | 0.28 |
| Mayo clinical subscore at IV steroids (9 vs ≤8) | 2.57 (0.64–10.30) | 0.18 |
| Mayo endoscopic subscore at IV steroids (3 vs 2) | NA | NA |
| UCEIS at IV steroids (8 vs ≤7) | 2.71 (0.73–10.10) | 0.14 |
| Hemoglobin at IV steroids (>105 g/L vs ≤105 g/L) | 0.36 (0.07–1.71) | 0.20 |
| PLT at IV steroids (>400×10^9^/L vs ≤400×10^9^/L) | 0.21 (0.03–1.68) | 0.14 |
| Albumin at IV steroids (>30 g/L vs ≤30 g/L) | 0.69 (0.19–2.57) | 0.58 |
| Erythrocyte sedimentation rate at IV steroids (>25 mm/h vs ≤25 mm/h) | 1.23 (0.26–5.93) | 0.80 |
| CRP at IV steroids (>65 mg/L vs ≤65 mg/L) | 2.25 (0.56–9.01) | 0.25 |
| Mayo subscore, stool frequency, at first IFX dose (3 vs ≤2) | 1.39 (0.35–5.55) | 0.64 |
| Mayo subscore, rectal bleeding, at first IFX dose (3 vs ≤2) | 11.40 (1.42–91.10) | 0.02 |
| Mayo subscore, physician’s global assessment, at first IFX dose (3 vs ≤2) | 1.98 (0.41–9.55) | 0.39 |
| Mayo clinical subscore, at first IFX dose (9 vs ≤8) | 2.72 (0.73–10.10) | 0.14 |
| Hemoglobin at first IFX dose (>70 g/L vs ≤70 g/L) | 0.29 (0.07–1.17) | 0.08 |
| PLT at first IFX dose (>350×10^9^/L vs ≤350×10^9^/L) | 0.32 (0.07–1.51) | 0.15 |
| Albumin at first IFX dose (>30 g/L vs ≤30 g/L) | 1.28 (0.35–4.78) | 0.71 |
| CRP at first IFX dose (>5 mg/L vs ≤5 mg/L) | 0.42 (0.11–1.68) | 0.22 |
| Hemoglobin after first IFX dose (>80 g/L vs ≤80 g/L) | 3.99 (0.50–31.90) | 0.19 |
| PLT after first IFX dose (>450×10^9^/L vs ≤450×10^9^/L) | 1.99 (0.50–7.99) | 0.33 |
| Albumin after first IFX dose (>30 g/L vs ≤30 g/L) | 1.70 (0.35–8.21) | 0.51 |
| CRP after first IFX dose (>20 mg/L vs ≤20 mg/L) | 1.78 (0.48–6.65) | 0.39 |
| Mayo subscore, stool frequency, at day 14 (3 vs ≤2) | 3.82 (0.78–18.60) | 0.10 |
| Mayo subscore, rectal bleeding, at day 14 (3 vs ≤2) | 5.71 (1.18–27.60) | 0.03 |
| Mayo subscore, physician’s global assessment, at day 14 (3 vs ≤2) | 11.20 (2.64–47.20) | 0.001 |
| Mayo clinical subscore, at day 14 (9 vs ≤8) | 16.50 (3.24–83.80) | <0.001 |
| Hemoglobin at day 14 (>90 g/L vs ≤90 g/L) | 0.90 (0.24–3.33) | 0.87 |
| PLT at day 14 (>450×10^9^/L vs ≤450×10^9^/L) | 1.99 (0.50–7.99) | 0.33 |
| Albumin at day 14 (>35 g/L vs ≤35 g/L) | 3.32 (0.89–12.40) | 0.07 |
| CRP at day 14 (>10 mg/L vs ≤10 mg/L) | 1.02 (0.27–3.79) | 0.98 |
| Concomitant use of wide-spectrum antibiotics | 1.97 (0.25–15.80) | 0.52 |
| Concomitant use of vancomycin | 4.66 (1.25–17.40) | 0.02 |
| Concomitant use of antiviral drugs | 1.88 (0.51–7.03) | 0.35 |

5-ASAs, 5-aminosalicylic acid; CRP, C-reactive protein; ESR, erythrocyte sedimentation rate; IFX, infliximab; IV, intravenous; NA, not applicable; PLT, platelet; UC, ulcerative colitis; UCEIS, Ulcerative Colitis Endoscopic Index of Severity; HR, hazard ratio; CI, confidence interval.

**Figure Legends**

**Supplementary Figure 1.** Details regarding the multiple imputation methodology. (A) See the pattern of missing data. (B) Perform the multiple imputation with R v.4.3.0, using a general linear model by pmm, and seed was set 20230526; five complete sheets were generated; the density distribution of original data and filled missing data were shown. (C) Select one single sheet for following analysis; perform univariate logistic regression analysis using clinical remission as dependent variable with original and five filled completed sheets; then select the sheet with the closest results to the original data. (D) Draw the density distribution for different variates to further verify the filled sheet was close to the original one. 5-ASAs, 5-aminosalicylic acid; Alb, albumin; BMI, body mass index; *C. diff*, *Clostridium difficile*; CMV, Cytomegalovirus; Cor, corticosteroids; CRP, C-reactive protein; ESR, erythrocyte sedimentation rate; Hgb, hemoglobin; IFX, infliximab; IV, intravenous; PLT, platelet count; UC, ulcerative colitis; UCEIS, Ulcerative Colitis Endoscopic Index of Severity.

**Supplementary Figure 2.** Receiver operating characteristic curves with accelerated induction as dependent variable. Cor, corticosteroids; CRP, C-reactive protein; ESR, erythrocyte sedimentation rate; Hgb, hemoglobin; IFX, infliximab; PLT, platelet count.

**Supplementary Figure 3.** Receiver operating characteristic curves with clinical remission at day 14 as dependent variable. Cor, corticosteroids; CRP, C-reactive protein; ESR, erythrocyte sedimentation rate; Hgb, hemoglobin; IFX, infliximab; PLT, platelet count.

**Supplementary Figure 4.** Receiver operating characteristic curves with colectomy at day 90 as dependent variable. Cor, corticosteroids; CRP, C-reactive protein; ESR, erythrocyte sedimentation rate; Hgb, hemoglobin; IFX, infliximab; PLT, platelet count.

**Supplementary Figure 5.** Flow diagram of literature enrollment of systematic review and meta-analysis. ASUC, acute severe ulcerative colitis.

**Supplementary Figure 6.** Funnel plot of systematic review and meta-analysis.
